# Real-time mixed reality display of dual particle radiation detector data

**DOI:** 10.1038/s41598-023-27632-1

**Published:** 2023-01-07

**Authors:** Oskari Pakari, Ricardo Lopez, Ivan Druckman, Emilee Meng, Erik Zhou, Ziang Wang, Shaun D. Clarke, Sara A. Pozzi

**Affiliations:** 1 Department of Nuclear Engineering and Radiological Sciences, University of Michigan, Ann Arbor, MI 48109 USA; 2 Department of Electrical Engineering and Computer Science, University of Michigan, Ann Arbor, MI 48109 USA

**Keywords:** Applied physics, Nuclear physics, Computer science

## Abstract

Radiation source localization and characterization are challenging tasks that currently require complex analyses for interpretation. Mixed reality (MR) technologies are at the verge of wide scale adoption and can assist in the visualization of complex data. Herein, we demonstrate real-time visualization of gamma ray and neutron radiation detector data in MR using the Microsoft HoloLens 2 smart glasses, significantly reducing user interpretation burden. Radiation imaging systems typically use double-scatter events of gamma rays or fast neutrons to reconstruct the incidence directional information, thus enabling source localization. The calculated images and estimated ’hot spots’ are then often displayed in 2D angular space projections on screens. By combining a state-of-the-art dual particle imaging system with HoloLens 2, we propose to display the data directly to the user via the head-mounted MR smart glasses, presenting the directional information as an overlay to the user’s 3D visual experience. We describe an open source implementation using efficient data transfer, image calculation, and 3D engine. We thereby demonstrate for the first time a real-time user experience to display fast neutron or gamma ray images from various radioactive sources set around the detector. We also introduce an alternative source search mode for situations of low event rates using a neural network and simulation based training data to provide a fast estimation of the source’s angular direction. Using MR for radiation detection provides a more intuitive perception of radioactivity and can be applied in routine radiation monitoring, education & training, emergency scenarios, or inspections.

## Introduction

Radiation imaging and source localization are essential tools in routine, emergency, or treaty verification scenarios involving radioactive materials. Imaging detector systems offer the ability to obtain the direction of incoming radiation, thus accelerating the search and enabling a search with less radiation exposure risk to the involved individuals^1^. A detector operator could hereby position themselves at a safe distance and observe the spatial distribution, or ’hot spots’, of radiation without the need to move the detector. Uncrewed vehicles such as drones or rovers could further reduce involved risks, as was demonstrated with the survey of radioactive accumulation sites at Fukushima nuclear power plant unit 1/2 exhaust stack^2^.

The handheld dual particle imager (H2DPI) developed at the University of Michigan^3^ is a portable fast neutron and gamma-ray detection system optimized for imaging purposes. Using a combination of double-scatter events in organic glass scintillators, photo-absorption in CeBr_3_, and pulse shape discrimination (PSD) for particle classification, the H2DPI is able to provide both gamma ray and fast neutron images simultaneously^4^. The H2DPI has been already used to image special nuclear material^3^, such as highly enriched uranium, ^252^Cf, as well as standard ($\alpha$,n) and gamma ray sources. Until now, radiation images and directional information are displayed on computer screens that project the data into 2D angular space, often in plots that convey intensity over the azimuth and altitude angles.

Mixed reality (MR) technologies that rely on head mounted devices are on the verge of widespread availability, including virtual reality (VR, fully virtual visual experience) and augmented reality (AR, overlay visuals on real world experience)^5^. MR has originally referred to the continuum of devices between AR and VR^6^, but more recent studies found no clear expert consensus on the exact definitions^7,8^. We use herein the definition of Mixed Reality as a technology that enables the interaction with virtual and real objects to display complex data in real-time as a holographic overlay to the user’s visual perception.

The MR smart glasses Microsoft HoloLens2^TM^^9^ is among the first more widely accessible devices that houses an array of sensors to map its surroundings and project holographic data into it. MR devices have found first applications that leverage the added virtual information, e.g. in medical applications such as surgery assistance^10^, anatomy training^11^, design visualization^12^, and radiotherapy^13,14^. A recent review on extended reality (XR) in spine medicine identifies a wide range of interest and needs for this type of technology^15^.

MR has also been explored in in the domain of work safety in construction^16^, radiation protection training^17–19^, as well as collaborative maintenance and facility management^20^ and city planning^21^. VR reconstructions of rooms with radiation sources have been used for remote inspections and source searches^22^. In a similar fashion, the use of AR and VR in light perception studies have shown promise in widening our understanding of human perception and optimize architecture^23^. Nonetheless, a recent study quantified the performance when using a VR device compared to computer monitors for a spatial understanding task^24^, and found no significant performance gain using VR. The envisioned benefits of using XR technologies are therefore not guaranteed.

MR has nonetheless the potential to be successfully applied in fields where a user may benefit from a direct visual representation of complex data (e.g. surgery, visualizing tumor locations), where timeliness of a signal may be important (e.g. in work safety, highlighting radiation zones or other safety hazards), or where effective education and training can benefit from a new way of representing the underlying concepts (e.g. radiation fields otherwise invisible to the human eye).

Radiation image interpretation requires the conversion of 2D angular information into 3D visual space. MR has the distinct advantage of eliminating this mental step, leading to a direct 3D impression of the radiation data onto the physical surroundings. In recent literature this specific application is, so far, unexplored - yet, as mentioned above, similar cases have been demonstrated in medicine and work safety. MR technologies could therefore enable non-rad-professionals (e.g., emergency responders, non-specialized armed forces, researchers in training) to perform routine radiation monitoring and respond to accident scenarios.

**Figure 1:**
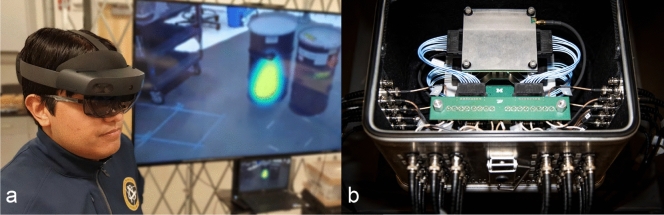
(**a**) Picture of a user wearing the Microsoft HoloLens2. The screen in the background depicts what the MR device shows the user - in this example an image of a hot spot over a barrel. (**b**) Picture of the (open) handheld dual particle imager (H2DPI), showing the top of the system.

The objective of this work is to demonstrate a real-time MR display of both gamma ray and fast neutron images created from H2DPI data in HoloLens2, as illustrated in Fig. 1. Hereby we integrate several cutting edge technologies, from silicon photomultiplier (SiPM) readout of novel organic glass scintillator, over an optimized data processing and network implementation, to 3D MR display of the data. Our open source implementation (see the Code availability statement below) is based on Unity, python, and Rust - all readily available code packages. Using Unity specifically implies that our solution is readily translatable to other platforms, such as smartphones. We showcase the MR view for different measurements using ^252^Cf spontaneous fission and ^137^Cs gamma ray sources, and qualitatively discuss the advantages and limitations of MR radiation visuals using the H2DPI.

## Results

### Radiation image calculation

**Figure 2:**
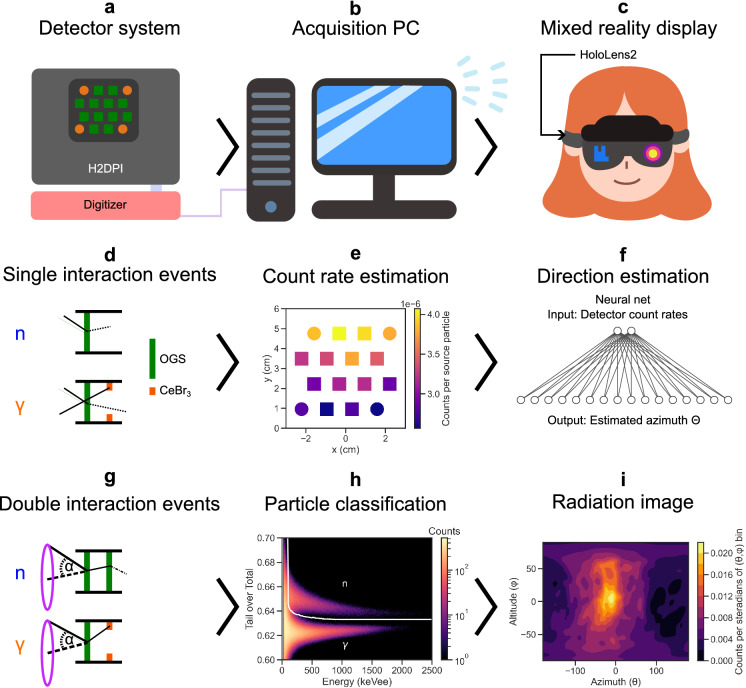
Overview of the data processing from particle interactions to MR display. (**a**) Radiation interactions in the H2DPI detector system are observed via SiPM arrays that are connected to a digitizer. (**b**) Acquired waveforms are sent to the acquisition PC in regular intervals over an optical wire, where they are filtered and sent over WiFi to the HoloLens2. (**c**) The HoloLens2 projects the data into the users field of view. Single events (**d**) can be used to estimate total count rates in each detector (i.e., both neutrons and gamma rays), which show a specific shape depending on the source’s azimuthal location (**e**). The average count rates per detector are given as inputs to a pre-trained neural network (**f**) that predicts the azimuth of the source, showing an arrow to the user. Double-scatter events in two different detector volumes (**g**) allow for the reconstruction of the detected particle’s incidence cone with opening angle $\alpha$. Subsequent particle classification of the individual events in the organic glass scintillator (OGS) via pulse shape discrimination (**h**) enables the differentiation between neutron-neutron and gamma-gamma incidence cones. A discrimination line between the pulse shape clusters (white) is found via fitting a double Gaussian into each energy equivalent slice and taking the valley as the decision threshold. Using a back projection algorithm the cones are overlaid to create the radiation image (**i**) in angular space (Azimuth $\Theta$, Altitude $\Phi$) for either gamma rays or fast neutrons. The user is then shown a 3D projection of the image in MR.

An overview of the data pipeline from detector interactions to MR display is shown in Fig. 2. The H2DPI system was set up on an optical board to ensure repeatable positioning, a more detailed description of the detector geometry can be found in the Methods section.

The H2DPI consists of an array of detectors optimized for imaging, notably through the arrangement of the lattice of 12 organic glass scintillator bars for fast neutron and gamma-ray detection and 8 CeBr_3_ inorganic scintillators for photoelectric gamma ray detection. The radiation images are calculated via double-scatter events in the H2DPI (Fig. 2g), i.e., all waveforms are filtered into event pairs that satisfy the conditions of having occurred within 30 ns of each other in two different detector volumes. These doubles events are then classified via the individual event waveforms into gamma ray or fast neutron events^4^ (Fig. 2h), and filtered to only consider neutron-neutron and gamma-gamma doubles. Each double-scatter event has an associated estimated incidence *cone*, which are superimposed to construct the so-called simple back projection image^3^. By using neutron-neutron or gamma-gamma events, cumulatively over time a neutron and/or gamma ray image can be calculated in angular space (Fig. 2i).

The digitized waveforms from the detector system (Fig. 2a) are sent in arbitrary batch sizes (typically set to write a batch roughly every 2 seconds) to the acquisition PC (Fig. 2b). The filtering of the events to double-scatter incidence cones is performed here. Individual cones are then available on a locally hosted server that the HoloLens2 accesses via the wireless network (Fig. 2c). Sending individual cones instead of images was found to reduce the overall network strain, allowing for a constant data stream from detector to HoloLens.

### Real-time radiation data display in MR

**Figure 3:**
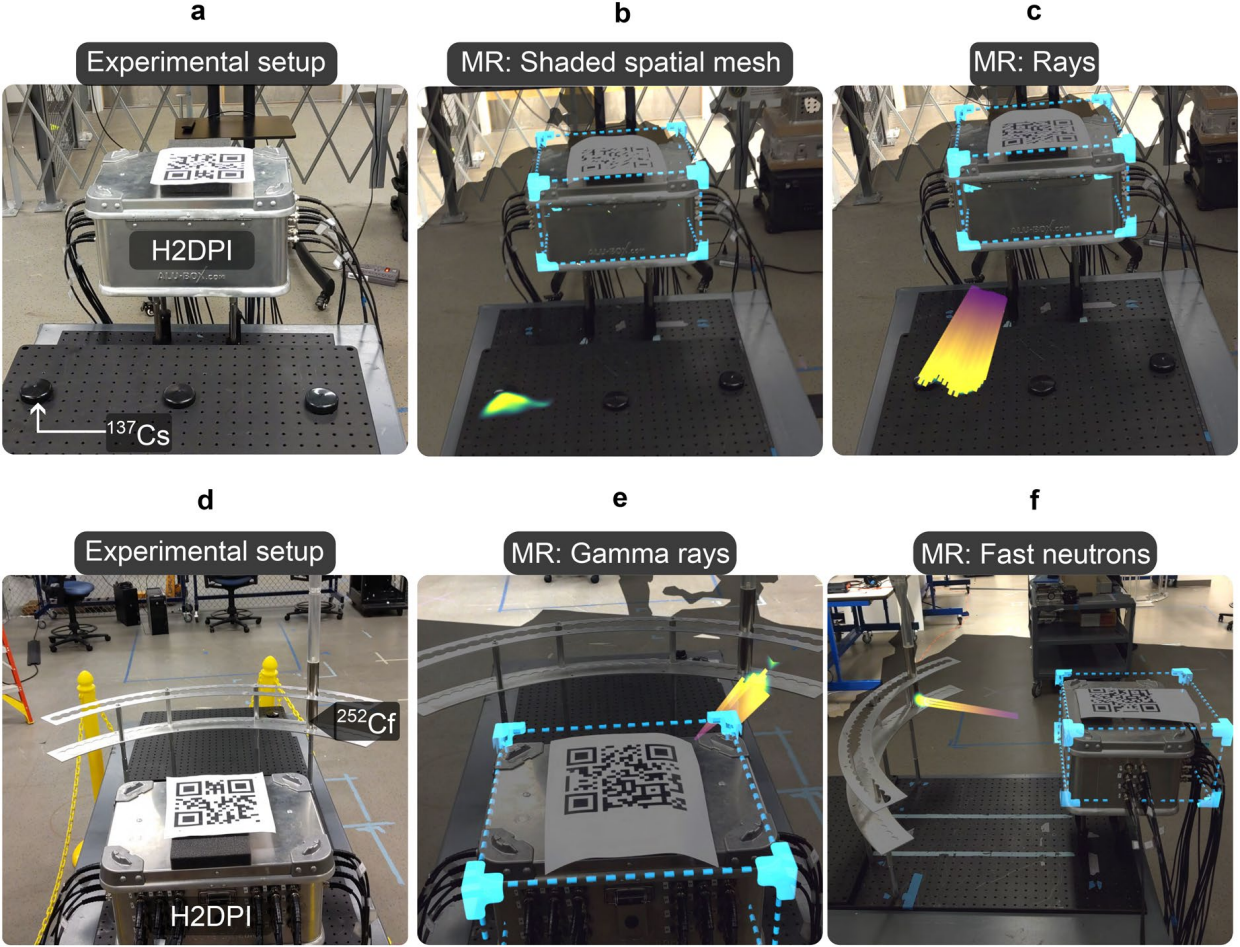
Examples of the mixed reality visuals as seen by the user wearing the HoloLens2. (**a**) A 100 $\mu$Ci ^137^Cs source is set under one of three caps in front of the detector system. (**b**) In MR, the user is shown a coloring of the spatial mesh after about 30 seconds of acquisition. (**c**) The user may optionally choose to also display rays that point to the hot spot. (**d**) A 1 mCi ^252^Cf source in front of the system. (**e**) The gamma ray image forms within approximately 20 seconds. (**f**) The user can switch to the (simultaneously acquired) neutron image via a wrist menu. A converged neutron image that intersects with the expected source location is seen after around one minute.

The MR display in the HoloLens2 is achieved via several steps using an application written in the Unity game engine^25^ that runs on the HoloLens. First the HoloLens is provided the location of the detector. 3D spatial mapping is a basic feature of the HoloLens, and an application programming interface (API) provides the spatial map of the surroundings to the back-end programmer. We further use the HoloLens API that allows the scanning of QR codes in space to localize a certain 3D coordinate in the given spatial map. A QR code is set on the surface of the H2DPI at a known position relative to the center of the detector system (see Fig. 3a,d), enabling the calculation of a reference location.

Simultaneously, the HoloLens uses the local WiFi network to query a server hosted on the acquisition PC to retrieve the detector data. After receiving the cones, the visualizer performs simple back projection to generate the radiation image texture in H2DPI space. A pixel shader is then used to render the image onto the spatial mesh. For each pixel on the spatial mesh, the pixel screen position is transformed into the reference coordinates, which are then transformed into H2DPI spherical coordinates, which are used to sample from the radiation image. A color map is applied to convey the intensity, creating a colored impression of the spatial mesh (that is transparent otherwise). A more intense coloring of the mesh would hereby be indicative of a more intense radiation image in that spatial direction.

On top of the spatial mesh coloring, we iterate through each pixel in the back projected texture and project a ray from the H2DPI to illustrate the angular nature of the information in the image. If the ray intersects with the spatial mesh, and exceeds a certain threshold, then it draws a line between the H2DPI and the spatial mesh. All of the mentioned processing steps typically require less than one second, thus providing a visually smooth experience. The display of either rays or spatial mesh coloring is available to the user through a virtual menu that is located on their wrist (i.e., when the user’s wrist is in the field of view of the HoloLens, a menu appears that has virtual buttons for settings).

As an illustration of the resulting visual experience, we display pictures taken with the HoloLens depicting two experiments in Fig. 3. In the first example, we hid a ^137^Cs source under one of three caps (Fig. 3a). After approximately 30 seconds of acquisition time, the HoloLens displays the colored spatial mesh (Fig. 3b) or rays (Fig. 3c) according to the back projection image. At any point, the user may toggle between the display of the gamma ray or neutron image. Examples of neutron and gamma ray images are shown in Fig. 3e,f, in which we display the MR view of an experimental setup using a ^252^Cf source set in front of the detection system.

An important aspect of the colored shading of the hot spot is the choice of colormap. We hereby followed the rule to use perceptually uniform sequential colormaps^26^. We implemented the standard perceptually uniform sequential colormaps used in Matplotlib (viridis, inferno, magma, plasma, cividis)^27^ as options, that the user can choose in the virtual menu. The user can therefore choose the appropriate colormap to maximize contrast for a given background, or to adjust for a user’s ability to distinguish colors. For instance, a colormap containing red (inferno) on a green background (e.g. grass) may not be informative to a user with deuteranomaly, and they may choose to switch to a yellow/blue based map (cividis).

### Fast source search via neural network

Double-scatter events are rare compared to single events, and in many scenarios a sufficiently converged image may not form within a given measurement time. In order to optimally guide the detector operator in moving the detector system to a closer location, we propose to use the single interaction count rates to predict the source’s location rapidly (Fig. 2d), as count rates can be estimated at any point in the measurement. Due to the arrangement of the detector lattice, we expect a gradient in the count rates (Fig. 2e, i.e., the detectors that are closer to the source have a higher count rate than the detectors further away, thus enabling a statistical learning method to predict the angle of incidence of the radiation).

The count rates per detector are estimated via a rolling average over the waveforms over a 1-2 second long measurement and given as input into a trained neural network that outputs an estimated azimuthal location of the source. We describe the neural network (training data and hyper-parameters) in the Methods section. In MR, the user is presented with an arrow pointing in the azimuthal direction of the prediction.

**Figure 4:**
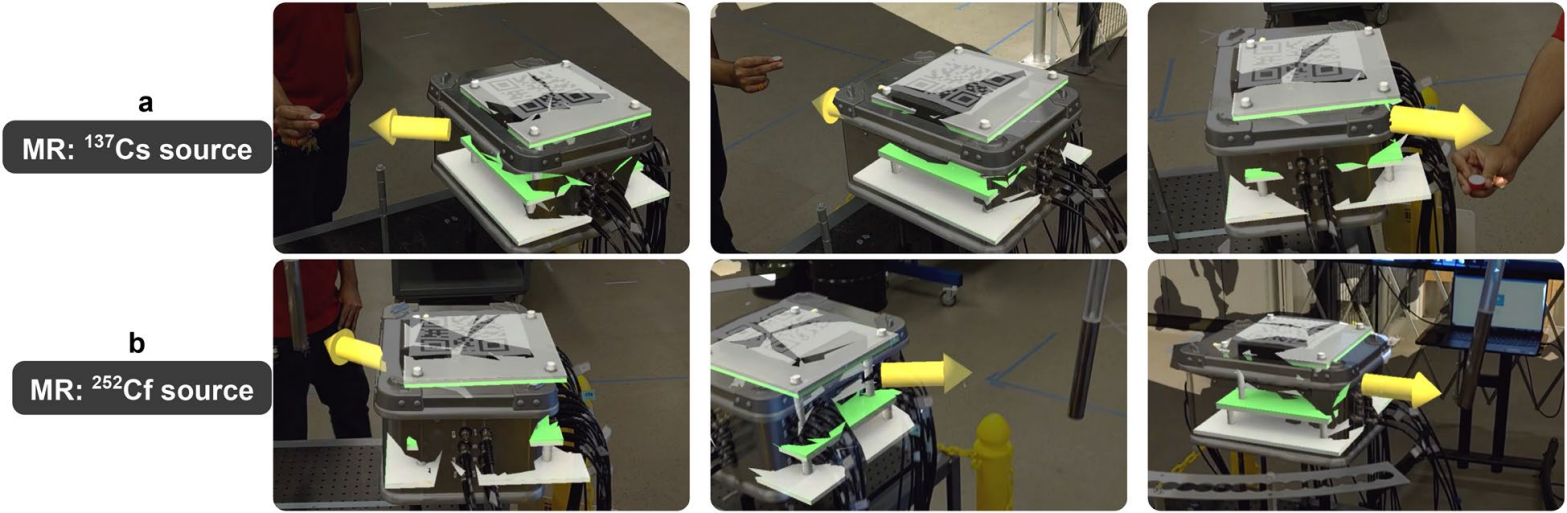
Examples of the mixed reality visuals as seen by the user wearing the HoloLens2 for the fast source direction estimate using a neural net. (**a**) A 100 $\mu$Ci ^137^Cs source is held in three different locations around the detector, the MR display shows a yellow arrow to represent the neural nets’ guess. (**b**) A 1 mCi ^252^Cf source is held at different locations around the detector. The prediction and display of the source’s estimated location took less than 1 second in every case.

Figure 4 shows an example of the MR visuals for the source direction estimation for both ^137^Cs and ^252^Cf sources. The prediction, due to the small size of the neural net, takes less than 1 second and provides the expected result. Uncertainty quantification of the neural network’s predictions are beyond the scope of this study, and we therefore present this result qualitatively as a proof of concept. Note that the neural network was trained on ^252^Cf simulations, but the predictions were correct for both ^252^Cf and ^137^Cs experiments. This shows promise for future investigations to elucidate the neural network performance, in particular with regards to training it on multiple sources, multiple distances, as well as the altitude angle.

### Detection limitations for the current H2DPI system

**Figure 5:**
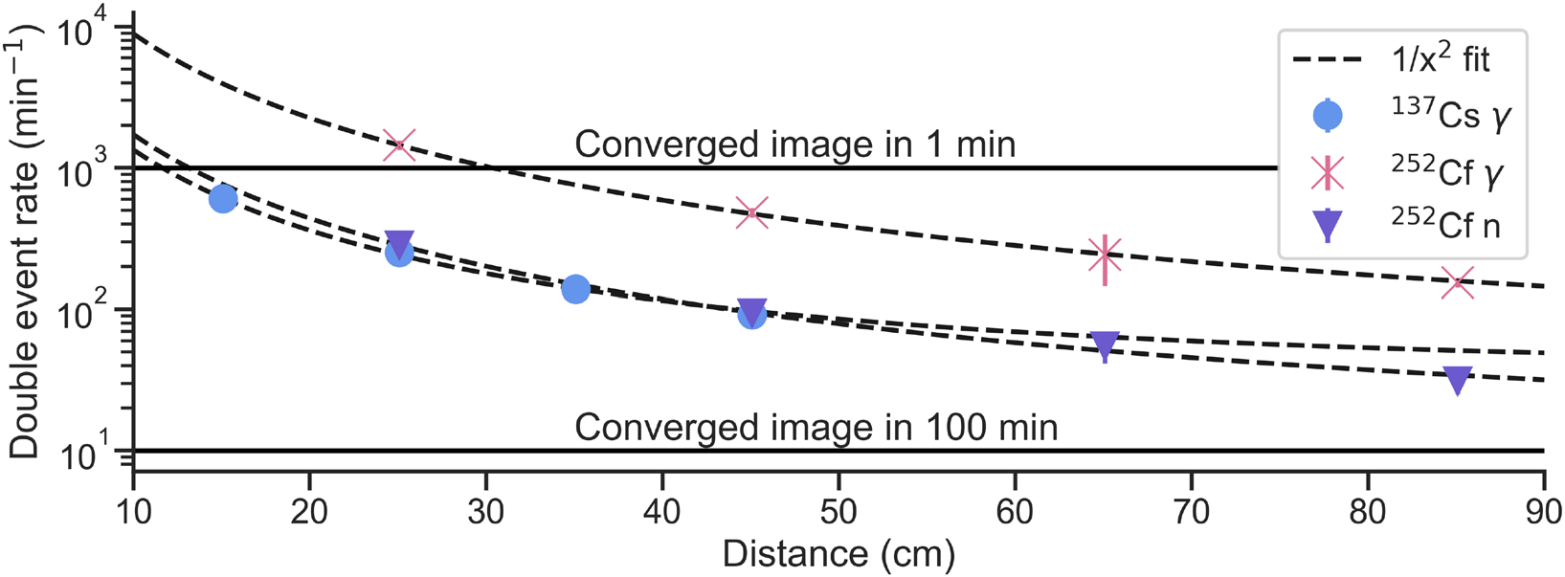
Double-scatter interaction rates (per minute) with distance of the source to the detector system for 100 $\mu$Ci ^137^Cs and 1 mCi ^252^Cf sources. Inverse square law model fits are shown for illustration, alongside two lines that indicate the time needed to achieve a converged image (1000 double-scatter events). The error bars are often smaller than the marker.

Double-scatter events are comparatively rare and their occurrence pose the main limitation to achieving a radiation image that is, by some empirically determined threshold, converged. In Fig. 5 we display the double-scatter event rates for ^252^Cf and ^137^Cs sources for increasing distances to the system to illustrate this. With increasing distance, as is obvious from the decrease in single event rate following approximately an inverse square law, we observe a decrease in double-scatter rates. We further indicate approximate event rates that would yield a converged image (arbitrarily defined as 1000 cones, based on the authors’ experience), showing that the used sources can only be imaged in less than 1 hour to convergence within a radius of 1 m around the system. The H2DPI system will in the future be upgraded to contain up to 64 organic glass scintillator bars, enhancing the efficiency of detection by approximately tenfold for neutrons and twenty-fold for gamma rays, alleviating the convergence time concern^4^.

The sources we used, however, are comparatively weak. An extrapolation of double event rates with activity is expected to scale linearly, thus allowing for predictions for specific scenarios. For example, most radiation accidents caused by lost or stolen sources in the late 20th century involved sources with activities >100 GBq^28^, thus being more than three orders of magnitude stronger than the sources we used in this work. A converged image would hereby, assuming the model fit we used for the gamma events from ^252^Cf, be formed within one minute at more than 10 meters distance, enabling a safe assessment of the source’s location even with the current system.

## Discussion

### Mixed reality: a better visualization tool?

The herein presented work is a proof of concept for the use of MR in radiation detection and imaging. We demonstrated the feasibility of using a hand-held detector to generate on-the-fly gamma ray and neutron images and source direction estimates in MR within seconds of their acquisition. This approach is achieved by using several cutting edge technologies in ensemble: a novel organic glass scintillator coupled to a silicon photomultiplier (SiPM) array for dual particle detection, a detector layout optimized for radiation imaging using double-scatter events, a neural network trained with Monte Carlo data to assist in the source localization, and finally MR visualization of the data using the Microsoft HoloLens2. We further explored two different visualization styles: a colored spatial mesh and optionally rays pointing towards the estimated source location.

We hereby improved on several aspects of recent literature: A previous study of using an array of NaI detectors for source direction estimation using a neural network showed high accuracy^29^, yet relied purely on gamma ray interactions and prediction was performed after the experiment. Our network was trained for both photons and neutrons and is able to provide a location estimation for either type of source in real-time. Other studies showed the MR visualization of radiation hot spots on a tablet device or AR using data from Geiger Mueller counters on a robot^30^ or a UAV^31^. We build upon these by demonstrating MR visualization in real-time, on a head mounted device, for both gamma ray and neutron detection. Recent developments for MR applications for general radiation protection education^32^ and operating room radiation awareness^17,33^ rely on transport calculations that are then translated to MR visuals - using real-time detector data may benefit from our proposed method and provide a more realistic representation of the radiation field. Our implementation is open source (see Code availability statement below) and based on a general 3D engine (Unity). This means that our implementation could be translated to other platforms that have basic MR/AR capabilities, such as smartphones or other smart-glass devices.

The main advantage of using MR visualization is the simplified interpretation of angular radiation images, i.e., not requiring the user to mentally project the 2D angular hot spots into 3D directions and plausible source locations. Radiation images can therefore now be interpreted by untrained individuals, impacting training requirements for general radiation measurements and allowing for flexibility in task delegation in time sensitive scenarios.

The MR visualization also conveys a more realistic size and uncertainty of the radiation images. In interpreting 2D radiation images, the authors themselves consistently underestimated the angular uncertainty when translating the images into a real space source location. We found that images that we previously perceived as converged turned out to be not sufficient to determine a source’s location once viewed in MR.

MR is, nonetheless, a subjective experience. Qualitatively speaking, we report an overall positive response to the use of the HoloLens2 in radiation laboratory demonstrations, pointing at worst to enjoyment from pure novelty, and at best to a truly more intuitive tool to visualize radiation. Future work therefore requires a more rigorous quantification of the improvements such as informativeness of the visualization, measured radiation exposure, and perceived improvement in workflow in the mentioned application scenarios. For instance, the subjective quality of perception could be evaluated using the Maryland Visual Comfort Scale^34^. The usefulness of VR/MR technologies should also be contrasted with psychological research on the concept of presence MR^35^ to elucidate why they are useful, thereby potentially aiding in the guided ideation for future applications of MR technologies.

## Methods

The authors affirm that the depicted individuals provided written consent for the publication of the figures and video used in this work.

### Handheld dual particle imager (H2DPI)

**Figure 6:**
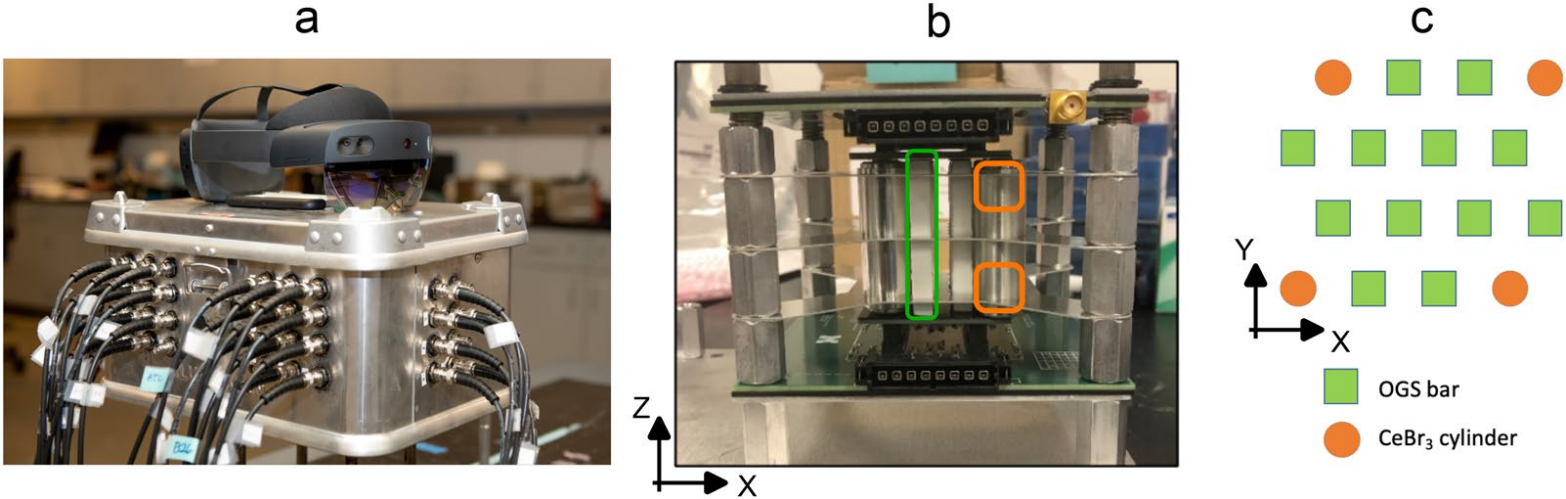
(**a**) Picture of the H2DPI with the HoloLens2 on top. (**b**) The detector volume of the H2DPI viewed from the side. (**c**) X-Y cross section schematic of the detectors of the H2DPI. The organic glass scintillator (OGS) bars are (50x6x6) mm^3^ in size. The CeBr_3_ detectors in the corners are cylindrical in shape (6x6 mm^2^) in height, set on both the top and bottom SiPM arrays. Each corner has therefore two CeBr_3_ detectors.

The H2DPI is a detector system developed at the University of Michigan that consists of an array of 12 organic glass scintillator bars, (50x6x6) mm^3^ each, and 8 CeBr_3_ scintillator cylinders, 6x6 mm^3^ each, as shown in Fig. 6. The detector arrangement is optimized for radiation imaging, using two silicon photomultiplier (SiPM) arrays (ArrayJ-60035-64P-PCB SensL) with (6x6x0.5 mm^3^) EJ-560 optical interfaces^3,36^ for light collection. The system notably uses dual ended readout and the relative light yields per pulse top versus bottom to estimate the z-position of a given interaction^37^. The H2DPI was connected via standard BNC and LEMO cables to two CAEN V1730S digitzer boards mounted to a CAEN VME8004 powered crate.

### Neural network for source direction estimation

The neural network was constructed using the TensorFlow 2.5 library in Python 3.8.8. Recently, the successful application of neural nets for this task was shown using arrays of NaI detectors^29,38^, or a single volume HPGe^39^. The neural network input nodes correspond to the count rates in each of the 20 detector volumes of the H2DPI. The output layer is the probability estimate for an azimuthal angle, here discretized by 1$^{\circ }$ increments into a vector of length 360. The input layer activation function was set to relu, whilst the output layer relied on softmax.

**Figure 7:**
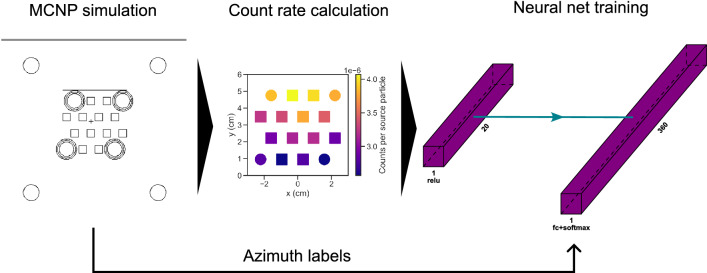
Overview of the training data generation for the source direction prediction using a neural net. 360 cases of a source around the detector were simulated using MCNP PoliMi, covering the azimuthal space in 1$^{\circ }$ increments. The count rates in each detector volume were then extracted alongside the known angular position of the source to create a training data set. The input nodes of the neural network correspond to the detector volumes, 12 organic glass scintillators and 8 CeBr_3_, i.e., a vector of length 20 with relu activation. The output layer of length 360 is fully connected (fc) to the input with a softmax activation, denoting the estimated probability for a source being at the index in degrees.

Training data was produced with the Monte Carlo particle transport code MCNPX-PoliMi^40^ that allows for both neutron and photon transport. An explicit model of the detector and casing was created, see Fig. 7. We simulated 360 source locations, each displaced by 1$^{\circ }$ increments at 30 cm distance to the detector, to cover the entire azimuthal range. We simulated each angular position with 10^9^ spontaneous fissions from a ^252^Cf point source. The 20 detector volumes were tallied for neutron and photon interactions and processed into count rates per detector volume using the post-processing code MPPost^41^.

The 360x20 data sets were then augmented by 100 by sampling Gaussian noise with zero mean and 10% of the counted particles as standard deviation to add noisy data to the training, thus yielding a data set of 36000x20. Note that the validation accuracy did not converge without the addition of the noisy data.

**Figure 8:**
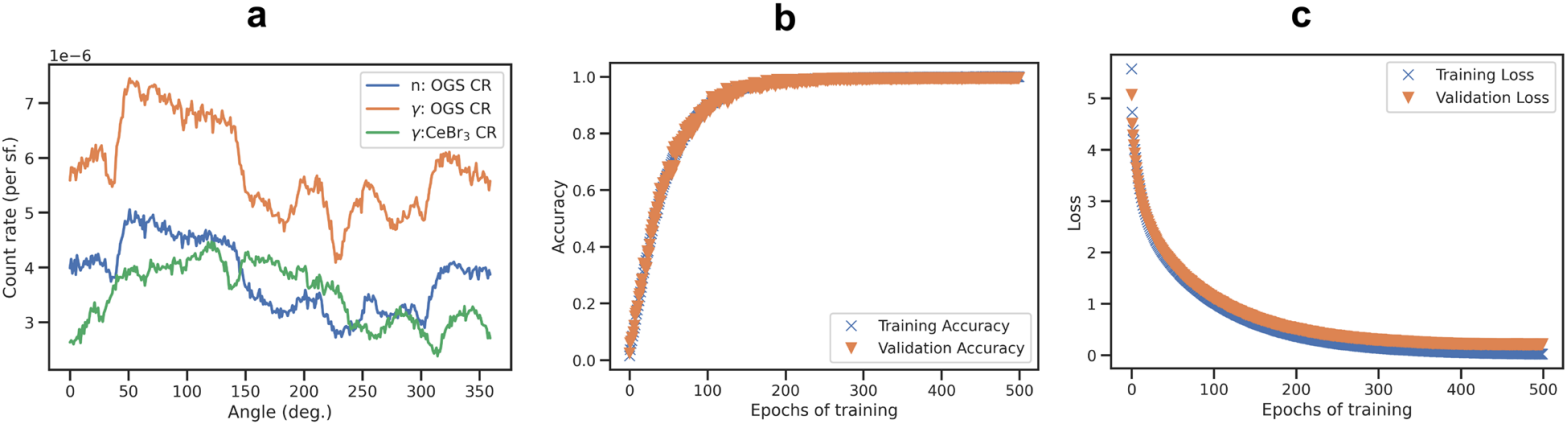
(**a**) Example of training data for neutron and photon interactions per spontaneous fission per source incidence angle in organic glass or CeBr_3_ detectors. (**b**) Training and validation accuracy with training iterations (’epochs’). (**c**) Training and validation loss with epochs.

Training was conducted using randomly shuffled training data, a 10% validation train split, and a categorical cross-entropy loss function. We display an example of the detector data with azimuth, as well as accuracy and loss of the model for both training data and validation data with training iterations (’epochs’) in Fig. 8. The model was trained at first for 1000 epochs, yet a possible overfit is seen in the validation loss increase above 400 epochs. The final model was thus trained for 300 epochs. The training and validation data accuracies increase with training iterations Fig. 8b) and overfitting is not observed. The final estimated validation accuracy was stated by the program as 98.8%. Note that adding hidden layers led to overfitting (i.e. a oscillating validation accuracy and validation loss increase with epochs^42^), which may require regularization if used.

The MR display uses the best estimate of the neural network (i.e., the output node with the highest value). For example, if node 90 has the highest value (typically >0.9), then the HoloLens will display a yellow arrow into the azimuthal direction 90$^{\circ }$.

## Supplementary Information

Supplementary Information.

## Data Availability

Data are available through reasonable request to the corresponding author (O. Pakari, pakari@umich.edu).

## Code availability

The code repository is publicly accessible under https://gitlab.eecs.umich.edu/umich-dnng/dnng-hololens-visualization.
